# “I don’t need a flat tummy; I just want to run fast” – self-understanding and bodily identity of women in competitive and recreational sports

**DOI:** 10.1186/s12905-018-0639-4

**Published:** 2018-09-03

**Authors:** Kirsten K. Roessler, Ashley E. Muller

**Affiliations:** 1 Department of Psychology Faculty of Health Science, University of Southern Denmark, Campusvej 55, 5230 Odense, Denmark; 2 Norwegian Center for Addiction Research, Institute of Clinical Medicine, University of Oslo, Postboks 1039 Blindern, 0315 Oslo, Norway

**Keywords:** Motivation, Competition, Self-understanding, Biography, Psychodynamic theory, Body image, Sports identity

## Abstract

**Background:**

Women who exercise intensively, whether competitive or recreational, devote a lot of time and energy into exercise, which requires high levels of ambition and motivation. The aim of the study is to investigate the self-understanding and bodily identity of different (competitive vs recreational) forms of exercise, and to investigate the role of important others (parents, siblings and social relations) for this self-understanding.

**Methods:**

A qualitative study using semi-structured interviews. An interactional psychodynamic framework informed the development of the interview questions focusing on the influence of their family, peers, and the meaning of exercise for their identity. Participants were recruited via local training centers and via the Danish Athletic Sports Association. A total of twenty highly physically active female athletes were interviewed, ten of whom participated in competitions (“competitve athletes”) and ten of whom did not (“recreational athletes”).

**Results:**

Self-related and social similarities and differences between competitive athletes and recreational athletes were found. Recreational athletes had supportive but not ambitious parents and used sport to reinforce their bodily self-efficacy and identity, while competitive athletes had highly engaged parents, especially fathers, and competed to externalize their identities as athletes. Correspondingly, the meaning of exercise was the activity itself, for recreational athletes, while competition was the means to the end of achievement, for competitive athletes.

**Conclusion:**

All athletes are affected and triggered by their biography and their environment. The biographical tradition of sport culture must be recognized as important for the engagement in different forms of physical activity in health and competition settings. If research can make the conflicts and relations of the self visible in sports culture, this could strengthen the recognition that the overall bodily well-being of athletes is important for women’s health.

## Background

Competitive athletes are sporting persons who are interested in outperforming other athletes. In this paper they consist of youth, adult and master members of the Danish national team in athletics (track and field). In Denmark, competition sports are organised by local clubs, and not by higher education institutions. Therefore, athletes are often dependent for many years on support from their parents. Competitive sports require a high degree of engagement, and athletes often have challenging lives. They have to meet their own ambitions, integrate time-consuming exercise into their daily lives, make decisions of how to combine career and education, and fulfil the high expectations of their surrounding networks, often represented by an ambitious trainer or a parent [1]. They often lack financial support for the time they spend with training and competition or have difficulties establishing a career after ceasing competitive sports [2].

There also exists a growing group of young women who exercise regularly and vigorously, but without engaging in competition. We refer to them hereafter as recreational athletes, although “recreational” perhaps belies the intensity with which many young women exercise. Competitive and recreational sportspersons have previously been investigated regarding cognitive aspects of motivation [1], especially with focus on their inner standards and self-evaluation [3], but women specifically have not been investigated. Women more so than men may be subject to intense pressure to use exercise as a way of meeting gendered bodily expectations such as weight and thin ideals [4, 5], and it is unknown whether such bodily motivation is related to the decision to exercise competitively or recreationally.

This paper will focus on the role of the self and of others relevant for exercise motivation. The self is hereby seen as a union of elements that constitute the identity of a person. We apply the interactional theory of the self of George Mead [6, 7], splitting the self in “me” and “I”. The “me” is the social self, taking in a set of attitudes of others as “me”, while the “I” is the individual response to the attitudes of others, for example, through familiar delegation [8]. Following Heinz Kohut, the self can only develop when its sense of worth and well-being is met in relationship with others. A person’s self is heterogeneous and comes into play in social activities as – in our case - in the field of sports and physical activity. Through taking in different social and interactional positions we differentiate the self.

Our interest takes its starting point from this interactive self-understanding. Which role is played by the interaction with other relevant people and siblings for the development of a competing versus a recreational self? In developing the desire to compete versus to exercise non-competitively but just as intensely, what impact does the feedback of others have, and the cohesion or the group climate? The psychoanalyst Stierlin [8] highlighted the parents as the most important “other”, writing that children are expected to overtake conscious and unconscious wishes or conflicts of their parents in the form of delegations, which can become central “missions” of individual family members throughout generations. Children may engage in competitive exercise because their parents have delegated this behaviour to them.

In sports psychology there exists a broad body of research on motivation, both for recreational and competitive athletes, and for men and women in sports in general [9], often outgoing from a more individual-centred cognitive psychological approach focussing on task versus ego motivation [10]. Texiera et al.’s [11] systematic review among all athletes found that intrinsic motivation predicted exercise behaviour more than fitness or body-related goals. Verkooijen and de Bruijn [12] more recently reported that a self-identity as an exerciser was a better predictor of exercise behaviour for female recreational athletes than male recreational athletes.

To our knowledge, the differences in self-understanding and bodily identity between intensive recreational and competitive sportswomen have not been investigated before, outgoing from a mostly interactional approach. However, interactional aspects can facilitate the comparison of these two environments for finding out the role of an important other for choosing your sports environment. When taking an interactional self-understanding as a point of departure, we would like to know how attitudes of the surrounding environment influence the athlete.

A recent cross-sectional study reported that highly motivated girls exercise more when their parents participate in the same activities, an association not seen among boys [13]. Psychodynamic theories may shed light on female motivation to exercise. Baxter-Jones et al. [14] hypothesized that women are more likely to compete if they have family members who themselves exercise regularly, value competition, and give positive feedback when the women exercise. If the women valued the rewards associated with competition, such as a desirable self-image or the feeling of being productive, they would additionally be more likely to compete. Such an understanding reinforces the importance of taking into account peer and family relationships and norms when investigating exercise behaviour [15]. Furthermore, the social contexts of the exercise environment, most visible in training groups, have the potential to foster interpersonal relationships and may also contribute to maintaining exercise [5, 16].

In this study, twenty high-intensity, highly motivated sportswomen were interviewed. Half were elite Danish track and field athletes (hereafter called “competitive athletes”), and half were sportswomen who did not compete, but exercised on a regular basis more than four times a week (hereafter called “recreational athletes”). Both competitive and recreational athletes devote many hours per week to exercise and have done so for many years, except one group chooses to compete and the other doesn’t.

The aim of this paper is to explore the self-understanding and bodily identity of different (competitive vs recreational) forms of exercise, and to investigate the role of important others (parents, siblings, and social relations) and the social context for this self-understanding.

## Methods

### Data collection and setting

The interview guide (see Table 1) was pilot tested and adjusted in order to ask questions in a more natural and meaningful way, and at the same time to allow the respondent to lead and form the conversation [17]. The interview was divided into four parts, asking for both individual and social aspects. Part I focussed on the participants’ *exercise background* by gathering descriptive data on former and current exercise or competition experience. Part II focused on the participant’s *motivation* to exercise or compete. Part III focused on the role of exercise in the *family*, and in the *social context*. We had specific interest in the parents’ and siblings’ exercise and competition behaviour, and in the feedback provided by their social environment (e.g. classmates). Finally, part IV examined participants’ *body image* and visibility in a sporting context. The interview guide finished with a question on the body image, and the athletes were asked how their bodies’ visibility when training or competing influenced their self-image, self-confidence and self-identity.

**Table 1:** Interview guide

Research question	Interview question
Part I: Exercise in your biography	How often do you exercise on a weekly basis? How old were you when you started to exercise? Which role did exercise play in your childhood? Which type(s) of exercise did you perform? Can you remember any emotions regarding exercise in your childhood? Did you have important friendships or other social relationships combined with your exercise? Did you prefer team or individual exercise? Did you like to compete?
Part II: Motivation for exercise	What is your main motivation for exercising? Did you have any benefits or disadvantages caused by your exercise? How did your social environment react to your exercise activity? Did your friends accept your sporting interest?
Part III: Exercise in your family and peer biography	Do you have siblings? Have your parents or siblings been active in sports or did they participate in exercise? Have you been playing games (e.g. ludo, chess) in your family? If yes, was winning an important issue? Do your friends or classmates have the same attitude towards exercise? Did this change during your life?
Part IV: Body image and visibility in a social context	Do you like your body? Has your relationship to your body changed over the years? How is it for you to compete and train public places, where other athletes or strangers can see your body? How much do you reflect about your body?

All interviews were conducted face-to-face, in an undisturbed setting. For the competition group, the interviews were mostly conducted in their training environments or in combination with observations under competitions, in three cases in their private homes. All recreational participants were interviewed at home. Participants were asked open-ended questions in a way that allowed them to answer reflectively, in turn giving the interviewer the possibility to ask follow-up questions in more depth. Interviews were conducted by the project leader and two master’s students in psychology and medicine. The first eight interviews were conducted by the project leader, while the remaining interviews were conducted by the master’s students trained by the project leader. The duration of the interviews was 30–60 min, and all were audio-recorded, transcribed, and analysed by the project leader in cooperation with the master’s student in psychology.

### Participants

Participants sought for this study had to be either competitive athletes, defined as currently or recently active in national teams of track and field, or recreational athletes, defined as exercising or practising recreational sports at least four times a week without competing during the last years. The only other inclusion criteria for participating in the study were being female, aged over eighteen, and Danish or Scandinavian speaking.

Competitive athletes were recruited via the Danish Athletic Sports Association (DAF). A member of DAF’s main office was identified as contact person and sent a list of potentially relevant competitive athletes to the project leader. The number of active Danish elite athletes on a high national or international level is quite limited, so the list included about 15 names of athletes who had represented Denmark on the youth or adult national team. The project leader contacted all athletes on the list, and ten agreed to participate. The project leader decided not to contact the two athletes participating in the Olympic Games due to problems of granting anonymity.

The recreational athletes were recruited by a written advertisement in two local fitness centres serving the local university population. The study was described to potential participants as an interview study on high-intensity athletes’ exercise habits and motivation. Twelve recreational athletes volunteered, but two were ultimately excluded because of participating in competitions during the last year aside from their primary recreational sport. Most of the recreational athletes had participated in some form of competitive sport in their childhood, as a part of after school occupation organised in local sports clubs. A high percentage of Danes are members of local sports associations when they are children, especially when they were brought up in the countryside.

Twenty sportswomen participated in the study, aged 18 to 53 (see Table 2).

**Table 2:** Characteristics of 20 competitive (C) and recreational (R) women

	ID	Age	Occupation	Exercise or sport (for C: years of competition experience)	Exercise environment
Competitive athletes	C1	23	Student	Running, hurdles (6)	With a trainer, in a group
	C2	30	Employed	Hurdles (24)	With a trainer, individually
	C3	20	Student	Running (4)	Trainer/group
	C4	20	Sabbatical	Running (6)	Trainer/group
	C5	28	Employed	Hurdles (20)	Trainer/group
	C6	19	Student	Running, hurdles (13)	Trainer/group
	C7	35	Employed	Running (15)	Trainer/group
	C8	18	Student	Running, long jump (13)	Trainer/group
	C9	22	Student	Running, hurdles (8)	Trainer/group
	C10	53	Employed	Heptathlon, high jump (25)	Trainer/individually
		Age	Occupation	Actual exercise or sport	Exercise environment
Recreational athletes	R1	24	Student	Running, cycling	No trainer/individually
	R2	27	Student	Badminton, pilates, other cardio	No trainer/individually and in a group
	R3	26	Student	Cycling, weight-lifting	No trainer/individually
	R4	28	Student	Cycling, weight-lifting, kickboxing	No trainer/individually and in a group
	R5	25	Student	Cycling, weight-lifting, yoga, other cardio	No trainer/individually
	R6	25	Employed	Running, cycling, weight-lifting	No trainer/individually
	R7	22	Student	Running, cycling, dance	No trainer/individually and in a group
	R8	22	Student	Running, cycling, weight-lifting, yoga	No trainer/individually
	R9	27	Student	Running, weight-lifting, dance	No trainer/individually and in a group
	R10	26	Student	Skiing, climbing	No trainer/individually

Characteristics of the participants and their exercise practices. *C* Competitive athlete, *R* Recreational athlete

The participants in the recreational group (*N* = 10) were in their twenties and were primarily college students within psychology and medicine, two highly selective university programs that require consistently high grades and achievement. The competitive group (*N* = 10) included both high school and university students (*N* = 7), and adult women working full-time (*N* = 3). Competitive athletes exercised either individually or in a group, but always supervised by a professional trainer. Their starting age for competition sport was between five and 14. Competitive athletes’ groups were club-based, with larger clubs having separate training groups per activity and smaller clubs combining athletes with different specialties. All members of the groups knew each other because they regularly exercised together. Recreational athletes also trained individually or in groups, but none had trainers. Their exercise groups were not static and varied from week to week (e.g. in a cycling class, the “group” was comprised of whoever had signed up that week). Competitive athletes ran or jumped in athletic events, while half of the recreational athletes ran longer distances. All reported at least one type of regular exercise.

#### Analysis of the interviews

The strategy for qualitative analysis was a systematic text condensation as a descriptive and explorative method, taking as a point of departure phenomenological analysis [18], in which the researcher attempts to accept the spoken meaning, but in addition searches for unexpressed, underlying meanings [19]. The procedure consists of the following steps: total impression - from chaos to themes; identifying and sorting meaning units - from themes to codes; condensation - from code to meaning; synthesizing - from condensation to descriptions and concepts [19].

All material was read with a pre-understanding as a clinical psychologist, with specific focus on internal conflicts and emotions, trying to find and describe certain patterns of interaction in the fields of competitive sports versus recreational sport. This methodology allows the researcher to work systematically with patterns expressed and to show stringency and discipline in data analysis without converting data to quantitative expressions such as generalising the results. The first author coded and analysed the tapes based on the above-mentioned question guide. To sustain the inter-coder reliability, the second author coded tapes individually, followed by the first author coding the tapes separately.

The transcripts were structured by questions, originally developed in psychodynamic interactional therapy to analyse group processes [20]. Here, we focused on the interrelation of relevant others (e.g. parents, siblings, coaches). Therefore, we differed the analysis after the following questions: Which themes mention the participants? How can we describe the interaction? Are there common rules or norms mentioned? Which emotions do the participants mention? Which emotions are visible? After the coding process, the main themes were described and analysed with a focus to describe patterns of emotional experiences showing relational aspects.

## Results

### Individual biography (part I)

In general, sport during childhood played a positive role in the biography of all athletes. Participating in non-school based leisure sports is a part of a typical Danish childhood, and all participants named many different sports activities, such as gymnastics, ball games, badminton, swimming, athletics or other sports. Participating in exercise was seen as a positive, healthy activity.

### Motives for exercising (part II)

The participants named a wide range of motives for exercising: to improve strength and speed, to improve health and well-being, and to be more physically active. Motives such as an interest in social interaction (for example, training together in the club environment) were mentioned occasionally, but seldom appeared as a primary expectation. The recreational athletes in particular were *not* looking for any private networks or social contacts, but were solely trying to improve their bodily strength and state of health.

### Parental influence (part III)

All interviewed participants in the recreational group, except one, were older sisters, and reported that exercising was a way to demonstrate healthy lifestyles and be role models for their younger siblings. The recreational athletes remember their parents as caring, interested, and supportive in their childhood athletic activities. It was noticeable that almost all participants showed the same emotion when talking about their parents. They smiled, had positive attitudes, and seemed to have relaxed relationships to their parents. It also seemed to be a common rule that the parents supported their children’s wish for exercise. Athletes who grew up outside of urban areas reflected on the time their parents had spent time driving them to different activities. However, parents mostly showed no specific preferences for a certain sport but were supportive in general.

“My family never took part in my decision for choosing a certain sport. I could start or stop whatever I liked to do. Probably, my brothers were influenced by my choice to choose football.” (R7).

“My father went riding, too. But he would never watch me when I was riding!” (laughs) (R4).

Only one participant described the praise of her father as her motivation for staying in competitive gymnastics when she was a child. Most of our participants came out of well-functioning families. Sport, even when not performed as competition sport, had the role of character-building.

“As children, we had to participate in a sport. Sport was seen as something positive and healthy. When we had chosen a sport, we should participate on a regular basis, even if we didn’t feel up to go to training a certain day; it was a way to learn discipline” (R5).

All parents were described in the same way: they liked their girls to be active and healthy, but the parents didn’t have a certain fondness for a certain sport. They neither seemed to have conscious or unconscious wishes for their children to be successful in sport. The interviewees seldom expressed negative emotions when referring to their childhood.

In the group of competitive women, the parents and siblings took another form of influence. The siblings were often described in relation to their physical achievement – for example if they had been faster in running or jumping. Half of them were eldest sisters, all having at least one sibling.

“Especially me and my sister, we have more *fighter genes* than most of other families; we always had to compete, even during holidays. (…) But we have always been happy for each other’ success. (…) But my age records are still better than hers.” (smiles)(C8).

For many of them, the parents had been part of “the club system”, knowing the training schedules, disciplines and environment. Their parents’ focus was so intense that, for some, it became very important to develop their own training routine and keep a certain distance from their parents’ wishes and considerations. In almost all interviews with competitive women, the fathers had been very engaged in their daughters’ sports careers. Sometimes the women enjoyed the fatherly attention and support, but often they had to stand up for themselves and get away from their fathers.

“My father asked my trainer: ‘How far can she make it? Which level will she reach?’ (...) and at a certain point I had to push him back and to say, ‘you have no idea what I am doing; please, stay out of my sports life.’” (C3).

For the recreational athletes, in contrast, parental interest in a specific form of exercise was not a part of their family context growing up. As most Danish children, they participated in extracurricular activities when they were aged eight to twelve years. And typical to girls in general, they tended to stop when reaching puberty. These women began exercising recreationally again after leaving their parents’ homes.

### Social settings (part III)

Another important motivational aspect was the possibility to be well liked for the competitive athletes, who often described themselves as lonely when children.

“I was not very popular at school, so sport gave me an identity, a niche!” (C2).

They named the sports club as an environment where they could “fit in” as girls, in contrast to their school environments, where they often felt as outsiders. When in their early twenties, they described themselves as ambitious and goal-oriented in contrast to their peers, who were more interested in parties and alcohol. They expressed their need to be part of a training group, where all were ambitious, and to form a larger network of social relations including a club, supportive parents, and training communities, which supported exercising.

A particular close relationship that was mentioned as important for many of the competitive women was with their (male) trainer:

“My first trainer (…) has known me since I was five years old, he knows exactly, how I am thinking, and he knows how to treat me, I trusted him, and I trained maybe seven times a week for 1.5 hours.” (C5).

This athlete (C5) described a dependent relationship to her trainer, a relationship that was an important part of her identity. For some competitive athletes, the trainer was part of the inner dialogue of an athlete (“he knows me better than my parent”) and became a person the athlete could approach at almost every time of the day. A close, supportive relationship to their trainer was expected, as were such relationships in their club. One participant felt unimportant to her trainer, and when she felt the environment in the training groups became too competitive and unhealthy, she lost motivation to compete.

None of the recreational athletes reported periods of isolation in school or other examples of unhappy social phases during childhood. In fact, social factors – either historical or current – were rarely mentioned as relevant to why exercised. Two of the recreational athletes mentioned that they liked the social atmosphere in their training environments. However, they preferred to exercise alone, even when participating in a group class, and did not report needing peer support to maintain their motivation.

### Bodily appearance and self-understanding (part IV)

The self is mentioned by all participants, both in the way they act towards others but also in the way they act towards themselves. Here, competitive athletes and recreational athletes sound almost identical: Exercising is making your “self” comfortable.

“It is great to exercise, you feel good about your*self*” (C1);

“I feel better about my*self*, when I have been exercising” (R7).

We also find a high interest in the bodily appearance – however, the recreational women use it as a kind of self-production, while the focus on the body for the competitive athletes is more a medium for interaction with other sport participants.

“If I have to look really good before going to a party, I push myself in the gym beforehand” (R7).

“I shave my legs the day before a competition; if I don’t, I will run slower, because I am heavier. I have also the ritual of fixing up my nails, as a form of competition preparation” (C8).

Taken at face value, these two quotes seem to show the same interest in the bodily appearance. However, in the first case the body is instrumentalised as a part of the self – when my body looks good, I look good, as I am my body. In the second case, the “preparation” of the body before a competition is part of a confidence-building ritual of the self. When I look good, I will run faster. So, here we find differences in the role of the self.

“I hate to train by myself; I need to compete with others under the training” (C1);

“I also like the way I look naked when I have been exercising.” (R7).

Both groups have a strong focus on their bodily appearance, though with different targets.

“The most important part was to become better at something, by myself.” (C5).

Rather than family roles or social influences, most recreational athletes were clearly focussed on their own appearances. They showed a high preoccupation with their bodies as embodied selves, differentiated in certain parts of muscles.

“Your self-worth gets better when you look into the mirror and think your abs are just great!” (R7).

Recreational athletes expressed a substantial need for body control in order to maintain their participation. Exercise was a tool to make them feel good about themselves physically, and they needed evidence of improvement – feedback in the form of more muscles and lower fat – in order to continue to exercise.

In addition to giving them physical self-confidence, exercise represented a “me-world”, a time slot belonging to themselves, without any influence of others.

“I am using my head the whole day (...), sometimes it is really nice to do something with your muscles.” (R1).

“[Exercise] is a distraction for me. To come into your body and out of your head.” (R8).

Using the body allows for mental relaxation, as one participant framed it. The meaning of exercising was placed in the self, but in two different ways. For the recreational group, the meaning of exercising was to combine movement with renewed energy, less stress, wellness, and relaxation. These effects could almost initiate an addiction to a physically active lifestyle, including everyday exercising.

The bodily self-understanding, i.e. “being in shape”, were not goals in and of themselves for competitive athletes but were tangential benefits. They were highly aware of being in shape, but in a non-narcissistic way more targeted to achievement than to their looks. The benefits of exercising that were the goals for recreational athletes were a by-product, or evidence, of their hard work which allowed them to be competitive.

For the competitive athletes, the meaning of exercising was threefold: firstly, a concrete specific event as goal (“I want to participate in the Olympic Games”, C8); secondly, an external view of themselves (“I get motivated when people come to see me”, C1); thirdly, a rejection and contempt of Danish youth life, including heavy alcohol consumption (“I think there should be more important things than just hanging around with friends, doing nothing”, C4). Participation in competitive sports implied benefits: it gave them a valuable meaning of how to organise their spare time in a meaningful way, and the positive reward of having success and being able to run fast.

For the competitive athletes, the meaning was more dependent upon their achievement in competitions. “I always wanted to be the best!” said C1. One could have good days or bad days, depending on their bodily performance. The competitive athletes described the importance of having an external goal, such as a certain championship or becoming part of the national team. They all agreed that it was less important to have a body shaped in a specific way than to have muscles, which they can use for achieving their goals.

“I would like to have that six-pack, but I can’t use it for my running. You are more focused on what you actually need. I would not profit from losing some pounds, I would lose muscles instead. Of course, I would enjoy less weight, but that would reduce my speed, so I don’t care. I don’t need a flat tummy; I just want to run fast!” (C1).

Another more unusual aspect in motivation was the role of overcoming injuries, not once mentioned by the recreational athletes. Every competitive athlete told about periods of injury when they hadn’t been able to train or to compete, sometimes followed by periods of psychological imbalance or depression; injuries often happened multiple times within an annual training cycle. Overcoming the injury and re-gaining the fitness lost was described as a motivation in and of itself.

#### Discussion and recommendations

We started our investigation by asking for individual differences in the self-understanding and bodily identity in two groups of exercising females. Our two groups differed regarding to age and exercise intensity, however, they show more similarities than differences regarding their socialisation, such as a low rate of parental divorce, a positive relation to their parents, and a positive attitude regarding exercise. For all women – exercising both competitively and recreationally – sport was an important part of their childhood life. All women exercised when they were children, often competitively as a part of their normal extracurricular activities, and especially in the rural areas, where club life is an important element of social participation.

When they exercised, they exerted and maintained a sense of self to themselves - exercise was thus an internal validation of their bodily self. We started our paper citing Mead’s definition of the sociality of the self, and Kohut’s psychodynamic way of defining narcissim. Following Kohut, narcissistic patients are suffering from “the realm of the self and of those archaic objects cathected with narcissistic libido (self-objects) which are still in intimate connection with the archaic self (i.e. objects which are not experienced as separate and independent from the self” [21]. The bodily fixation of the recreational athletes has in the result section been shown as dangerous for a healthy self-object, as all energy would go to the bodily self. This fixation seemed to be the case for one women who had developed an eating disorder, usually a risk in elite sports [22], and continued to exercise up to twelve times a week.

The recreational group defined exercise in narcissistic manners, as being in contact with an inner or real mirror. They were using bodily activity to find a perfect balance between academic achievement and female identity. The way they utilized their bodies had an important meaning for their self-experience as healthy and capable young women. Using the “strong is the new skinny” ideal they defined themselves as high-achieving, goal-oriented, modern women. This finding aligns with previous reports of exercise in order to meet gendered expectations in a sample of low-income clinical patients [5]. Unique to the competitive group was that competition allowed them to project, or externalize, their identities. They took less interest in having a perfect body, but focused on external goals, such as participation in a certain level of competition, a new personal best or becoming part of the national team.

Narcissism as a theme in sport was mentioned by Roberts et al. [23], who investigated the role of narcissism in the motivation of female rugby players. They found that athletes with higher scores in narcissism competed better, while athletes with less narcissism had problems in mastery-focused climates.

An interesting aspect of the study was the difference in the bodily self-understanding regarding the wholeness of the body. Both groups talked surprisingly often about isolated parts of their body (for example arms, abs or thighs). But while the recreational athletes mostly focussed on these parts as expression of a lack of perfection, the competitive athletes were more worried about the functions of these body parts. When running 100 or 200 m, thighs are extremely vulnerable. Both groups can focus on their thighs, but while one group is concentrated on their form and appearance, the other group looks at their function. The almost nervous concentration and fixation on the body parts of competitive athletes is here more of caring character. Competitive athletes describe and understand their bodies like mothers their children – always slightly worried, listening and looking, but also proud. Here, the narcissistic fixation aims at achievement, while the recreational body covers the danger of a loss of perfection.

The question remains, in which way can a psychodynamic view contribute to sport research and health? This study might be understood as a supplement to recent studies on the culture of sports [24], especially with focus on the existential aspects [25]. Aspects of meaning and authenticity in the bodily understanding may deepen the understanding of psychological process.

All investigated women showed several similarities, such as spending a lot of time exercising and a certain degree of dependency on physical activity for retaining a stable bodily identity. Physical exercise and bodily control seemed here to give the recreational athletes especially a feeling of control over life in general, sometimes in the risk zone for getting injured or developing exercise addiction [26, 27]. But the investigation of the aspect of competition revealed psychological differences, which can be divided into patterns regarding the development of the self, meaning, and parental involvement. More than twenty years ago, Fortier et al. [28] reported that competitive athletes demonstrated less intrinsic motivation than recreational athletes. In contrast, both groups in our study showed a high intrinsic motivation, but with different inner psychic backgrounds. The recreational athletes were absorbed by the shape of their bodies; they identified with a culture where the control of the body shows a control of the self [29]. The difference between our findings and Fortier et al. may be one of time. Women are nowadays subject to increasingly rigorous body ideals (e.g. low fat percentage, lean muscle) and lifestyle ideals (intensive physical activity) propagated by current gendered trends such as “fitspiration” [30], and the recreational athletes seem to have internalized these ideals to a larger extent than the competitive athletes. Thin ideals propagating extreme thinness may be more harmful to female recreational athletes than athletic ideals lauding lean muscle [31]. Here, several of the competitive women stressed the point of not being focussed on a specific bodily ideal, even if they all seem to have a preference for flat stomachs.

The most challenging difference was the dynamics of parental delegation. Parental delegation aims at owning the inner life of a child in an intrapersonal and interpersonal way. Parental delegation can hinder the successful inner ownership of goals and attitudes. A healthy development requires three conditions: a capacity for self-object differentiation, a tolerance of ambivalence, and a sense of integrity having a cohesive ego [8]. This inner ownership can be pathological when it is transferred to the family level, such as in a situation of parental over-owning. In our case study, numerous women in the competitive group named explicit examples of over-owning, where the parents delegated their own ambition to their daughters, over-identified with her sports career by being present all the time, asked the trainer for background information, and gave practical tips. Knowing that sports psychology has focussed on the aspect of perfectionism [32, 33], familial delegation could explain a part of it. Alongside the danger of being a part of their parents’ ambitions, most of the competitive athletes underlined the immense emotional, practical and financial support they received from their families.

Our findings regarding motivation are different to those reported by Healey et al. [34], who described an autonomous drift motivating competitive athletes. We show that both competitive and recreational sportswomen are driven and triggered by their surroundings. Their motivation is not always an owned story of themselves. Knowing that athletic achievement is produced not only by a single athlete, but also by an environment, it was astonishing that the club environment seldom appeared as a motivational factor in the interviews [16].

## Conclusions

When producing knowledge for the health benefit of the athletes and sports environments, it will be important to include research that reveals their practices as seen from their internal and self-oriented perspective. If athletes do not find meaning in physical activity or competition, they will not give priority to investing time and energy in it. If research can make the conflicts and relations of the self visible in sports culture, this could strengthen the recognition that the overall bodily well-being of athletes is important.
